# Supplementary material for: Seroprevalence of Diphtheria and Tetanus Immunoglobulin G among the General Health Population in Guangzhou, China
Source: Vaccines (Basel). 2024 Apr 4;12(4):381. doi: 10.3390/vaccines12040381 (PMC11053562; doi:10.3390/vaccines12040381)
Supplement: Supplementary file 1 [file vaccines-12-00381-s001.zip › vaccines-2925645-supplementary.pdf]

Supplemental Table S1. The diphtheria and tetanus toxoid IgG-specific antibody levels by doses and sex.

Doses	No. (%)		Diphtheria GMC (IU/ml, 95%CI)		P	Tetanus GMC (IU/ml, 95%CI)		P
	Female	Male	Female	Male		Female	Male	
0	1(0.39)	6(1.96)	-	0.023(0.007,0.078)	<0.05	-	0.01(0.002,0.042)	>0.05
1	6(2.34)	3(0.98)	0.096(0.031,0.294)	0.097(0.006,1.631)	>0.05	0.121(0.014,1.044)	0.499(0.085,2.919)	>0.05
2	4(1.56)	4(1.31)	0.021(0.001,0.362)	0.18(0.064,0.502)	>0.05	0.033(0.001,1.728)	0.387(0.114,1.312)	>0.05
3	46(17.97)	35(11.44)	0.137(0.101,0.187)	0.128(0.076,0.215)	>0.05	0.353(0.234,0.531)	0.214(0.12,0.381)	>0.05
4	136(53.13)	178(58.17)	0.125(0.106,0.146)	0.202(0.173,0.236)	<0.01	0.347(0.275,0.437)	0.214(0.12,0.381)	<0.01
5	63(24.61)	80(26.14)	0.161(0.13,0.199)	0.223(0.175,0.285)	<0.05	0.229(0.165,0.319)	0.332(0.246,0.45)	>0.05
	256(100)	306(100)						

Supplemental Table S2. The diphtheria toxoid IgG-specific antibody levels by doses and area.

Doses	No. (%)			Diphtheria GMC (IU/ml, 95%CI)			P
	Central urban	Suburban	Outer suburban	Central urban	Suburban	Outer suburban	
0	2(1.11)	0(0)	5(2.79)	0.15（0,50000）	-	0.023(0.004,0.117)	>0.05
1	2(1.11)	2(0.99)	5(2.79)	0.102(0,5808430)	0.228(0.011,4.595)	0.067(0.027,0.162)	>0.05
2	0(0)	5(2.46)	3(1.68)	-	0.096(0.017,0.558)	0.03(0,6.121)	>0.05
3	27(15.00)	21(10.34)	33(18.44)	0.28(0.167,0.469)	0.103(0.061,0.175)	0.085(0.06,0.122)	<0.01
4	121(67.22)	105(51.72)	88(49.16)	0.247(0.204,0.299)	0.142(0.123,0.164)	0.111(0.088,0.14)	<0.01
5	28(15.56)	70(34.48)	45(25.14)	0.418(0.266,0.658)	0.159(0.136,0.188)	0.161(0.116,0.224)	<0.01
	180(100)	203(100)	179(100)				

Supplemental Table S3. The tetanus toxoid IgG-specific antibody levels by doses and area.

Doses	No. (%)			Tetanus GMC (IU/ml, 95%CI)			P
	Central urban	Suburban	Outer suburban	Central urban	Suburban	Outer suburban	
0	2(1.11)	0(0)	5(2.79)	0.041(0,1620897)	-	0.01(0.002,0.066)	>0.05
1	2(1.11)	2(0.99)	5(2.79)	0.029(0,547.15)	0.029(0,547.15)	0.238(0.025,2.247)	>0.05
2	0(0)	5(2.46)	3(1.68)		0.176(0.02,1.521)	0.055(0,87.853)	>0.05
3	27(15.00)	21(10.34)	33(18.44)	0.381(0.239,0.608)	0.374(0.232,0.603)	0.187(0.094,0.372)	>0.05
4	121(67.22)	105(51.72)	88(49.16)	0.72(0.594,0.872)	0.355(0.285,0.442)	0.288(0.207,0.401)	<0.01
5	28(15.56)	70(34.48)	45(25.14)	0.269(0.181,0.401)	0.319(0.239,0.425)	0.241(0.145,0.4)	>0.05
	180(100)	203(100)	179(100)				
